# Prevalence of Overweight and Obesity among Students in the Kumasi Metropolis

**DOI:** 10.1155/2015/613207

**Published:** 2015-02-11

**Authors:** D. B. Kumah, K. O. Akuffo, J. E. Abaka-Cann, D. E. Affram, E. A. Osae

**Affiliations:** Department of Optometry and Visual Science, College of Science, Kwame Nkrumah University of Science and Technology, Kumasi, Ghana

## Abstract

The aim was to determine the prevalence of obesity and overweight among students in the Kumasi metropolis. In a descriptive cross-sectional study, 500 students aged 10 to 20 years were examined from two junior high schools selected by multistage sampling technique and three randomly selected senior high schools. Height and weight were measured in all participants and the body mass index (BMI) of each individual was calculated. Body mass index classes were calculated according to the International Obesity Task Force standards. Out of the 500 students, 290 (58.00%) were males and 210 (42.00%) were females. The prevalence of underweight, normal weight, overweight, and obesity was 7.40%, 79.60%, 12.20%, and 0.80%, respectively. Overweight was more prevalent among students than obesity. There is therefore the need to establish effective public health promotion campaigns among students in order to curtail future implications on health.

## 1. Introduction

Obesity and overweight have both been described as anomalous accumulation of excessive body fat which may be harmful to health [1]. There is no single cause to explain all cases of obesity and overweight but most studies implicate imbalance in the amounts of calories consumed and those expended [1]. Energy breakdown is said to be less than energy buildup. The disruption of the normal satiety feedback mechanisms, hyperinsulinism, insulin resistance, and genetics are some of the biophysiological causes of obesity and overweight [2].

Some researchers also attribute obesity and overweight to obesogenic environments where people are frequently exposed to and consume savory foods with hidden fats and sugars that can impair metabolism and lead to obesity [3]. Some public health experts also associate the development of obesity and overweight with socioeconomic status, urban lifestyle, family size, physical inactivity, educational status, cultural factors, and poor eating habits [4–6]. Persons who spend their leisure inactively such as in prolonged watching of television and playing of video games have been said to be at risk of obesity [7].

In addition to being pathologically chronic, obesity and overweight have been associated with other comorbidities in both younger and older populations. Heart disease, vision problems, cancer, hepatic impairment, diabetes, and other disease conditions as well as the economic burden involved in managing these conditions have been associated with obesity and overweight [8, 9]. Obese and overweight individuals are also stigmatized; in some societies, people portray a disease stigma towards those suffering from obesity and overweight and see them as immoral, lazy, unclean, and voracious [10].

While the majority of the researches done highlight obesity and overweight as problems of the developed countries, recent studies also show that the third world countries are no exception [11, 12]. Reports show that the issue of childhood and adolescence obesity in third world countries requires public health attention [12, 13]. During the time of this research, there were limited published findings on childhood and adolescence obesity and overweight in Ghana.

This study aimed to determine the prevalence of obesity and overweight among students aged 10–20 years in the Kumasi metropolis of Ghana, to sensitize the public on the emerging trend of childhood and adolescence obesity and overweight in Ghana and to provide data for public health professionals and policy planners.

## 2. Methods

### 2.1. Sampling

A descriptive cross-sectional study of 500 students aged 10 to 20 years was carried out from May 2010 to June 2010 in the Kumasi metropolis, Ghana. Simple random sampling was used to select three senior high schools (SHS). Multistage sampling technique was used to select 2 junior high schools (JHS). Simple random sampling was used to select one submetropolis from a total of 10 submetropolises. Within the selected submetropolis, simple random sampling was used to select two junior high schools. In each selected school, one class out of each educational level was randomly selected. Students included in the study were apparently healthy.

### 2.2. Sample Demographics

Kumasi is an urban and cosmopolitan settlement. Therefore, students recruited for the study came from diverse ethnic backgrounds; most of them were Ashantis and others belonged to represented minor groups from Northern Ghana. The study respondents were largely from middle class homes. Parents of the recruited students had little or no formal education and worked as tradesmen and tradeswomen and skilled professionals.

### 2.3. Anthropometric Measurement

Height and weight were measured using standardized protocols [14]. Weight was measured without shoes to the nearest 0.1 kg using a single previously standardized portable weighing scale. Height was measured without shoes and recorded to the nearest 0.1 cm with a height rod fixed on a wall. The body mass index (BMI) of each individual was calculated as weight in kilograms divided by height in metres squared. The BMI classes we adopted are the same as those used by the International Obesity Task Force for the international overweight and obesity cutoffs for children [15, 16].

### 2.4. Data Analysis

The statistical package for social scientists (SPSS) software, version 16.0, Chicago, USA, was used to analyze the collected data. Descriptive statistics were used to examine anthropometric characteristics. Independent *t*-test was used to test gender differences in anthropometric characteristics.

### 2.5. Ethical Consideration

This research was conducted with approval from the heads of the selected junior and senior high schools and from the teachers. Informed consent forms were signed by parents to approve the participation of their wards.

## 3. Results and Discussion

A total of 500 students aged 10 to 20 years took part in the study. Two hundred and ninety (58.00%) males and 210 (42.00%) females were included in the study. The overall mean age was 15.91 ± 1.78 years (16.00 ± 2.00 years for the males and 15.00 ± 2.00 years for the females).

Table 1 shows a summary of the measured anthropometric parameters per sex of study subjects. Males were significantly heavier and taller than females (*P* < 0.05) but there were no differences in BMI (*P* > 0.05).

The overall prevalence of underweight, normal weight, overweight, and obesity was 7.40%, 79.60%, 12.20%, and 0.80%, respectively. Among males and females, prevalence of overweight was 6.80% and 5.40%, respectively, while the prevalence of obesity was 0.20% and 0.60%, respectively. These results are slightly close to those reported in a study done among students in Nigeria [17] where there was a high prevalence of overweight and a low prevalence of obesity.

Comparable to other studies done in Ghana and other second world countries [18, 19], this study showed that the prevalence rate of overweight was higher among female students than male students. There are however divergent findings in studies done in developed nations [20, 21] where prevalence rates of overweight among male study subjects were higher than among females. For this study, the difference in the prevalence of obesity and overweight between the genders was insignificant (*P* > 0.05); the apparently higher values for males could be confounded by a greater number of male respondents (290) as compared to the female respondents (210).

While challenges to our study design did not allow for understanding reasons for the distribution of obesity and overweight among the genders, other studies report cultural attributions as to why females may be more overweight or obese than their male counterparts [17]. A study showed that obese females considered putting on weight as a sign of affluence and happiness. Others also believed that being obese afforded the needed strength in their sport and accorded them the necessary respect [22].

This is supported by undocumented reports from some areas in Ghana, where excessive weight gain is considered to be the result of eating good and healthy food.  Table 2 summarizes the prevalence of underweight, normal weight, overweight, and obesity according to the gender of the students.

Findings from this study must be considered in the light of certain challenges to the study design. The study depended on anthropometric measurements to establish whether or not the students were of the following categories: underweight, normal weight, overweight, or obesity. A study conducted in Ghana extensively looked at how the various weight statuses were linked to socioeconomic status of subjects and level of education of students' parents [23]. Another study also found significant associations between smoking and overweight and obesity [17] while other reports reveal positive associations between some psychosocial factors (e.g., loneliness and social isolation) [23, 24].

Even though this study did not look at socioeconomic status, smoking, dietary factors, loneliness, behavioural patterns, age of menarche, and order of births of the students and how these may influence their different weight statuses, it is reasonable from other studies [17, 23–26] to say that a complex interplay of these factors could contribute to our findings in this study.

To the best of our knowledge, the study has provided an insight into the prevalence of obesity and overweight among children and adolescents aged 10–20 years in the Kumasi metropolis of Ghana.

## 4. Conclusion

The study revealed a high prevalence of overweight (12.20%) among the students. Therefore, there is a need to establish effective prevention and health promotion programmes among the students. This would enable maintaining healthy weights and avoiding the possible immediate and long-term health complications associated with overweight and obesity.

## Figures and Tables

**Table 1:** Summary of anthropometric measurements per sex of study subjects.

Parameters	Males	Females
	Mean ± SD [95% CI]	Mean ± SD [95% CI]
Weight (kg)	54.71 ± 9.27 [53.64, 55.78]	51.12 ± 10.36 [49.71, 52.52]
Height (cm)	160.85 ± 9.03 [159.80, 161.89]	157.24 ± 8.97 [156.02, 158.46]
BMI (kg/m^2^)	21.04 ± 2.64 [20.74, 21.35]	20.57 ± 3.27 [20.13, 21.02]

CI: confidence interval; BMI: body mass index.

**Table 2:** Prevalence of underweight, normal weight, overweight, and obesity according to gender.

Prevalence rate (%)					
Gender	Underweight	Normal weight	Overweight	Obesity	Total
Male	2.60	48.20	6.80	0.20	**57.80**
Female	4.80	31.40	5.40	0.60	**42.20**
Total	**7.40**	**79.60**	**12.20**	**0.80**	**100.00**
